# B7-H3-targeting Fc-optimized antibody for induction of NK cell reactivity against sarcoma

**DOI:** 10.3389/fimmu.2022.1002898

**Published:** 2022-10-07

**Authors:** Ilona Hagelstein, Monika Engel, Clemens Hinterleitner, Timo Manz, Melanie Märklin, Gundram Jung, Helmut R. Salih, Latifa Zekri

**Affiliations:** ^1^ Clinical Collaboration Unit Translational Immunology, Department of Internal Medicine, German Cancer Consortium (DKTK), University Hospital Tuebingen, Tuebingen, Germany; ^2^ Cluster of Excellence iFIT (EXC 2180) “Image-Guided and Functionally Instructed Tumor Therapies”, University of Tuebingen, Tuebingen, Germany; ^3^ Department for Immunology and German Cancer Consortium (DKTK), Eberhard Karls University, Tuebingen, Germany; ^4^ Department of Medical Oncology and Pneumology (Internal Medicine VIII), University Hospital Tuebingen, Tuebingen, Germany

**Keywords:** sarcoma, B7-H3, mAb, Fc-optimized, immunotherapy, NK cells

## Abstract

Natural killer (NK) cells largely contribute to antibody-dependent cellular cytotoxicity (ADCC), a central factor for success of monoclonal antibodies (mAbs) treatment of cancer. The B7 family member B7-H3 (CD276) recently receives intense interest as a novel promising target antigen for immunotherapy. B7-H3 is highly expressed in many tumor entities, whereas expression on healthy tissues is rather limited. We here studied expression of B7-H3 in sarcoma, and found substantial levels to be expressed in various bone and soft-tissue sarcoma subtypes. To date, only few immunotherapeutic options for treatment of sarcomas that are limited to a minority of patients are available. We here used a B7-H3 mAb to generate chimeric mAbs containing either a wildtype Fc-part (8H8_WT) or a variant Fc part with amino-acid substitutions (S239D/I332E) to increase affinity for CD16 expressing NK cells (8H8_SDIE). In comparative studies we found that 8H8_SDIE triggers profound NK cell functions such as activation, degranulation, secretion of IFNγ and release of NK effector molecules, resulting in potent lysis of different sarcoma cells and primary sarcoma cells derived from patients. Our findings emphasize the potential of 8H8_SDIE as novel compound for treatment of sarcomas, particularly since B7-H3 is expressed in bone and soft-tissue sarcoma independent of their subtype.

## Introduction

Sarcomas are malignancies of mesenchymal origin with relatively rare occurrence that are classified depending on the tissue origin (1, 2). They comprise more than 100 distinct subtypes with different biological behavior which eventually results in differing responses to treatment (3, 4). Five-year survival rates of 60–80% have been reported for patients undergoing surgical resection with subsequent chemotherapy (5). Outcome for patients with metastatic disease at time of diagnosis or for patients with recurrence of disease is considerably worse (6, 7). Even though therapeutic options have significantly increased over the last years, there is an urgent need for new treatment approaches that are desperately needed to improve patient outcomes (8, 9).

Treatment outcome in various types of cancer has been significantly improved by introduction of immunotherapy with monoclonal antibodies (mAbs). Prominent examples are Herceptin and Rituximab, which are now established standard treatment options for patients with Her2 expressing breast cancer and B-cell non-Hodgkins lymphoma, respectively (10, 11). Nevertheless, there is still plenty of room for improvement regarding the efficacy of so far available tumor-targeting mAbs, and in many disease entities including sarcoma no immunotherapeutic mAbs are yet available. Antitumor mAbs elicit therapeutic efficacy to a substantial part by induction of antibody-dependent cellular cytotoxicity (ADCC). The major effector cell population which in humans mediates this fundamental mAb function are natural killer (NK) cells (12, 13). To reinforce therapeutic efficacy of tumor-targeting mAbs, modification of the antibody Fc part is one possible approach. Affinity to the Fc receptor CD16 on NK cells can be increased by genetically engineering the glycosylation motifs or the amino-acid sequence of the Fc part. This Fc optimization potentiates the capability of mAbs to engage Fc receptor expressing immune effector cells like NK cells (14, 15). To engraft mAb treatment for additional disease entities, identification of suitable target antigens that are widely expressed on tumor cells while ideally being not expressed on healthy cells is inevitable. In sarcomas, this is particularly challenging due to lack of well-established target antigens, among others because of differences between the many subtypes (8).

B7-H3 (CD276) belongs to the B7 protein family and is classified as an integral transmembrane protein (16–18). So far, the immunoregulatory role of B7-H3 is still under discussion, alike its role in cancer pathogenesis (19–21). Expression of B7-H3 has been reported for a multitude of human cancers which include glioma, acute myeloid leukemia (AML), lung adenocarcinoma, ovarian cancer, neuroblastoma, pancreatic cancer, and also certain sarcomas, whereas it is largely absent in healthy tissues (22–27). In addition, B7-H3 is expressed on the tumor vasculature in many cancers (28–30). Overexpression of B7-H3 is linked to unfavorable disease course and poor prognosis for patients (25, 31, 32) and has been suggested to impair antitumor reactivity of T cells and NK cells (19, 29, 33). Based on these findings, B7-H3 appears to be an attractive target antigen for immunotherapy, as (i) B7-H3 expression is almost exclusively limited to tumor tissue, (ii) expression on tumor vasculature allows for additional targeting of both cancer cells and vasculature, allowing for dual mode of anticancer action and (iii) blocking of B7-H3 might reduce its immunosuppressive properties. Accordingly, several B7-H3-directed therapeutics are presently under investigation.

In the present study, we report on the overexpression of B7-H3 in various sarcoma subtypes and the development of an Fc-optimized B7-H3 mAb, which was characterized with regard to its potential to induce ADCC of NK cells against sarcoma cells.

## Material and methods

### Cells

Isolation of healthy donor peripheral blood mononuclear cells (PBMC) was carried out by density gradient centrifugation (Biochrom, Berlin, Germany). PBMC were collected from healthy donors of mixed age and gender and randomly selected, for each experiment. Cryopreserved cells were cultured at 37°C overnight in media prior the use in functional experiments. In all cases, written informed consent, in accordance with the Helsinki protocol, was given. The study was conducted according to the guidelines of the local ethics committee.

The sarcoma cell lines RD-ES (rhabdomyosarcoma), SaOs (osteosarcoma), SW1353 (chondrosarcoma), SW872 (liposarcoma) and SW982 (synovial sarcoma) were purchased from ATCC (American Type Culture Collection) and cultured as previously described (34). Cell lines used in experiments were cultivated for a maximum of two months. To validate cell authenticity, the respective immunophenotype provided by the supplier was examined. To exclude contamination of cultured cells with mycoplasma, cells were tested regularly every three months. Patient-derived sarcoma cells from patients diagnosed with liposarcoma, chondrosarcoma, rhabdomyosarcoma, osteosarcoma or synovial sarcoma were obtained from outgrowth cultures of resected primary tumors as previously described (34).

### Production of antibodies

Generation of 8H8_SDIE and 8H8_WT as well as corresponding controls was carried out by chimerization (human immunoglobulin G1/κ constant region) of the anti-B7-H3 mAb 8H8 and control mAb MOPC21, respectively and Fc-optimization (S239D/I332E modification) for mAbs as described previously (35). Briefly, respective light chain (LC) and heavy chain (HC) plasmids were received using the EndoFree Plasmid Maxi kit from Qiagen (Hilden, Germany) as described in the manufacturer’s instructions. For antibody production, the ExpiCHO cell system (Gibco, Carlsbad, CA) was used according to the manufacturer’s recommendations. mAbs were purified from media by protein A affinity chromatography (GE Healthcare, Chicago, IL) followed by preparative size exclusion chromatography (HiLoad 16/60 Superdex 200; GE Healthcare). To ensure quality and purity of produced antibodies, analytical size exclusion chromatography (Superdex 200 Increase 10/300 GL; GE Healthcare) and 4–12% gradient SDS-PAGE gels (Invitrogen; Carlsbad, CA) was performed using the gel filtration and Precision Plus standard from Bio-Rad (Hercules, CA), respectively.

### Expression of B7-H3 mRNA based on TCGA database analysis

Data on relative expression of B7-H3 mRNA for tumor tissue and normal tissue samples was obtained from the Cancer Genome Atlas (TCGA) database and the GTEx project utilizing the Gene Expression Profiling Interactive Analysis (GEPIA) web server as described previously (26). Data sets for 5 different tumor subtypes (275 tumor/349 normal tissue) colon adenocarcinoma, (286/60) kidney renal papillary cell carcinoma, (179/171) pancreatic adenocarcinoma, (486/338) lung squamous cell carcinoma, and (262/2) sarcoma samples were downloaded from TCGA (http://www.oncolnc.org) and analyzed employing the online web server GEPIA (http://gepia.cancer-pku.cn).

### Pcr

B7-H3 primers were QuantiTect Primer Assay Hs_CD276_1_SG (Qiagen), GAPDH primers were 5′-AGCCACATCGCTCAGACAC-3′ and 5′-GCCCAATACGACCAAATCC-3′. 1-2 x 10^6^ cells were used for total RNA isolation utilizing the High Pure RNA Isolation Kit (Roche, Basel, Switzerland) followed by cDNA synthesis using FastGeneScriptase II (NIPPON Genetics Europe, Düren, Germany) as described in the manufacturer’s instructions, respectively. Reverse transcriptase–polymerase chain reaction (RT-PCR) was performed as described previously (36, 37). Quantitative PCR (qPCR) was performed using PerfeCTa SYBR Green FastMix (Quanta Biosciences Beverly, MA) with a LightCycler 480 (Roche) instrument.

### Flow cytometry

For analysis of B7-H3 surface expression, fluorescence-conjugates of B7-H3 mAb or isotype control (Biolegend, San Diego, CA) were used. For dose titration and binding experiments, cells were incubated with 8H8_WT, 8H8_SDIE, Iso_WT and Iso_SDIE followed by anti–human PE conjugate (Jackson ImmunoResearch West Grove, PA).

To stain CD16 positive NK cells, fluorescence-conjugates CD3-APC/Fire, CD14-BV785, CD16-APC, CD19-FITC and CD56-PECy7 (all from Biolegend) were used.

Quantitative analysis of immunofluorescence to determine the number of B7-H3 molecules on the cell surface was performed using a murine B7-H3 Hybridoma-derived antibody and the QIFIKIT (Dako, Hamburg, Germany) as described previously (35).

For staining of intracellular IFNγ, cells were stained with CD3-FITC and CD56-PECy (both from BioLegend) followed by fixation and permeabilization and staining with mouse anti-human IFNγ-BV421 (clone b27) in 1:25 dilution using the Fixation/Permeabilization Solution Kit with BD GolgiPlug from BD Biosciences according to manufacturer’s instructions.,

Flow cytometry-based determination of target cell lysis was conducted as previously described (34). In brief, sarcoma cells were loaded with 2.5 μM CellTrace™ Violet cell proliferation dye (Thermo Fisher Scientific, Waltham, MA) prior to seeding in cocultures with PBMC of healthy donors in the presence or absence of the antibodies (1 μg/mL each). Measurement of equal assay volumes was allowed by using beads (Sigma). The percentage of living target cells was calculated as follows: 7-AAD^-^ cells upon treatment/7-AAD^-^ cells in control × 100.

7-AAD (BioLegend) staining (1:200) was used to exclude dead cells from flow cytometric analysis or LIVE/DEAD™ Fixable Aqua (Thermo Fisher Scientific). All samples were analyzed using the BD FACS Canto II or BD FACSCalibur (BD Biosciences). Data analysis was performed using FlowJo software (FlowJo LCC, Ashland, OR).

### Analysis of NK cell activation and degranulation

To determine activation and degranulation of NK cells within healthy donor PBMC, 20,000 sarcoma cells or 5,000 patient-derived sarcoma cells were cocultered with PBMC (E:T ratio 2.5:1) in the presence or absence (untreated) of treatment (1 μg/mL). For analysis of degranulation, Brefeldin A (GolgiPlug, BD Biosciences) was added into the coculture. Cells were harvested after 4 h and stained for CD107a expression followed by flow cytometric analysis. After 24 h, cells were harvested and stained for CD69 expression followed by flow cytometric analysis. Analysis of CD25 expression was performed after 72 h by FACS analysis. NK cells were selected as CD3^-^ CD56^+^ cells within PBMC.

### Analysis of cytokine expression and secretion

For analysis of cytokine secretion, healthy donor PBMC were cultured with 20,000 sarcoma cells or 5,000 patient-derived sarcoma cells (E:T ratio 2.5:1) with or without (untreated) the indicated mAbs (1 µg/mL each). After 24 h, coculture supernatants were analyzed for secretion of Granzyme A, Granzyme B, Perforin, Granulysin, sFasL, TNF, IL-2, IFNγ, IL-4 and IL-10 by Legendplex assays (BioLegend) according to the manufacturers protocol. For analysis of intracellular cytokine expression, PBMC were handled as described above and cultured for 4 h in the presence of Brefeldin A (GolgiPlug, BD Biosciences) and Monensin (GolgiStop, BD Biosciences). After incubation, cells were stained for flow cytometry-based analysis. NK cells were selected as CD3^-^ CD56^+^ cells within PBMC.

### Analysis of target cell lysis

Cytotoxicity of PBMC against sarcoma cells was analyzed by BATDA Europium assays after 2 hours as described previously (38). Percentage of specific lysis was calculated as follows: 100 × [(experimental release) – (spontaneous release)]/[(maximum release) – (spontaneous release)].

To perform long-term cytotoxicity analyses, the IncuCyte^®^ S3 Live-Cell Analysis System (Essenbioscience, Sartorius, Göttingen) was used. Sarcoma cells were seeded in 96-well plates and cocultured with PBMC of healthy donors (E:T ratio 5:1) with or without the indicated mAbs (1 µg/mL each). To determine the confluence of sarcoma cells, images were taken with 10x magnification every 4 h. To quantify living cells, confluences were normalized to the respective measurement at T=0 h. Cell confluence at T=0 h was set to 100%.

### Statistical analysis

Data are represented as mean ± standard deviation of replicates or individual values. Statistical analyses were performed utilizing the GraphPad Prism software (version 9). Significant differences were calculated using the Student’s t tests, one-way ANOVA, nonparametric Mann–Whitney test, or log-rank test. P values are represented as: *p < 0.05.

## Results

### B7-H3 is expressed in bone and soft-tissue sarcoma independent of subtype

**Figure 1:**
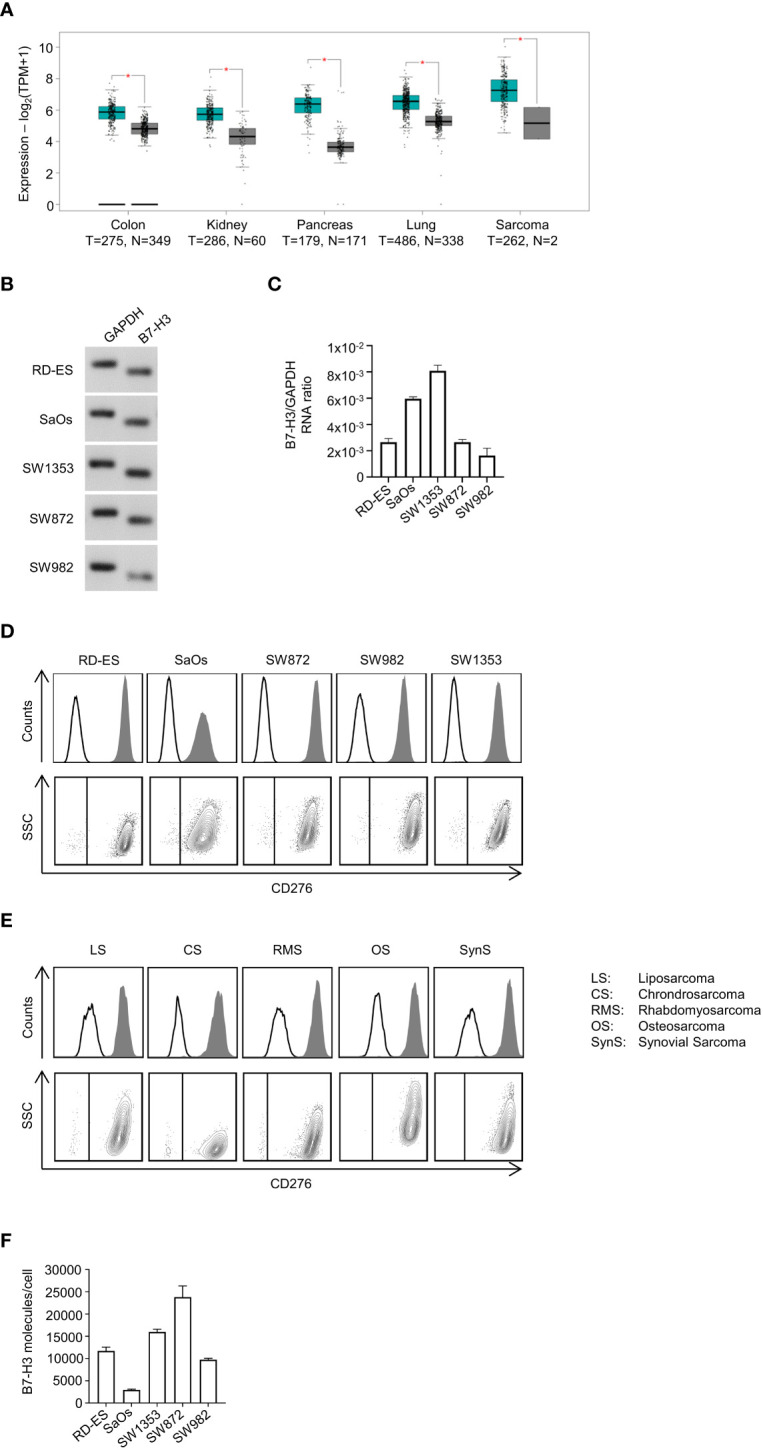
Characterization of B7-H3 expression in sarcoma cell lines and patient-derived sarcoma cells. **(A)** Relative mRNA expression of B7-H3 in indicated tumor and corresponding normal tissues was analyzed using the online web server GEPIA. T, tumor tissue; N, normal tissue **(B)** B7-H3 mRNA expression of sarcoma cell lines RD-ES, SaOs, SW1353, SW872 and SW982 was determined *via* RT-PCR with GAPDH serving as control. PCR products were visualized by agarose gel electrophoresis. **(C)** mRNA expression analysis of B7-H3 mRNA relative to GAPDH mRNA in five different sarcoma cell lines. Results for n=3 experiments are shown. **(D, E)** Surface B7-H3 expression on the indicated sarcoma cells was analyzed by flow cytometry using mAb against B7-H3 (shaded peaks) and corresponding isotype control (open peaks). Exemplary histograms (upper panels) and dot plots (lower panels) from one representative experiment of a total of three with similar results are shown. B7-H3 expression of **(D)** sarcoma cell lines and **(E)** patient-derived sarcoma cells of different subtypes dissociated from primary tumor samples are shown, respectively. **(F)** B7-H3 molecule counts on sarcoma cell lines were determined by FACS. Results for two independent experiments are shown.

So far, B7-H3 expression has been reported for many solid tumors (39), but little is known regarding its expression in the multiple subtypes of bone and soft-tissue sarcoma. As a first step, B7-H3 mRNA expression level data sets derived from TCGA of tumor and corresponding normal tissues were analyzed for relative B7-H3 expression. Analysis included data sets for 275/349 (tumor/normal tissue) colon adenocarcinoma, 286/60 kidney renal papillary cell carcinoma, 179/171 pancreatic adenocarcinoma, 486/338 lung squamous cell carcinoma, and 262/2 sarcoma. Compared with RNA expression in normal tissues, the expression of B7-H3 was profoundly increased in all analyzed data sets (**Figure 1A**). Based on these findings, we analyzed expression of B7-H3 mRNA in rhabdomyosarcoma (RD-ES), osteosarcoma (SaOs), liposarcoma (SW872), synovial sarcoma (SW982) and chondrosarcoma (SW1353) cells. Ubiquitous expression of B7-H3 with varying expression intensity in all tested soft-tissue and bone sarcoma cell lines was observed (**Figures 1B, C**). As a next step, we studied surface expression of B7-H3 on sarcoma cells. FACS based analysis of different sarcoma cell lines and patient-derived sarcoma cells derived from multiple subtypes revealed a varying extent of B7-H3 surface expression (**Figures 1D, E**). B7-H3 molecule counts were found to range between 2,960 (SaOs) and 23,797 (SW872) (**Figure 1F**).

### Generation and characterization of Fc wildtype and Fc-optimized B7-H3 mAbs

**Figure 2:**
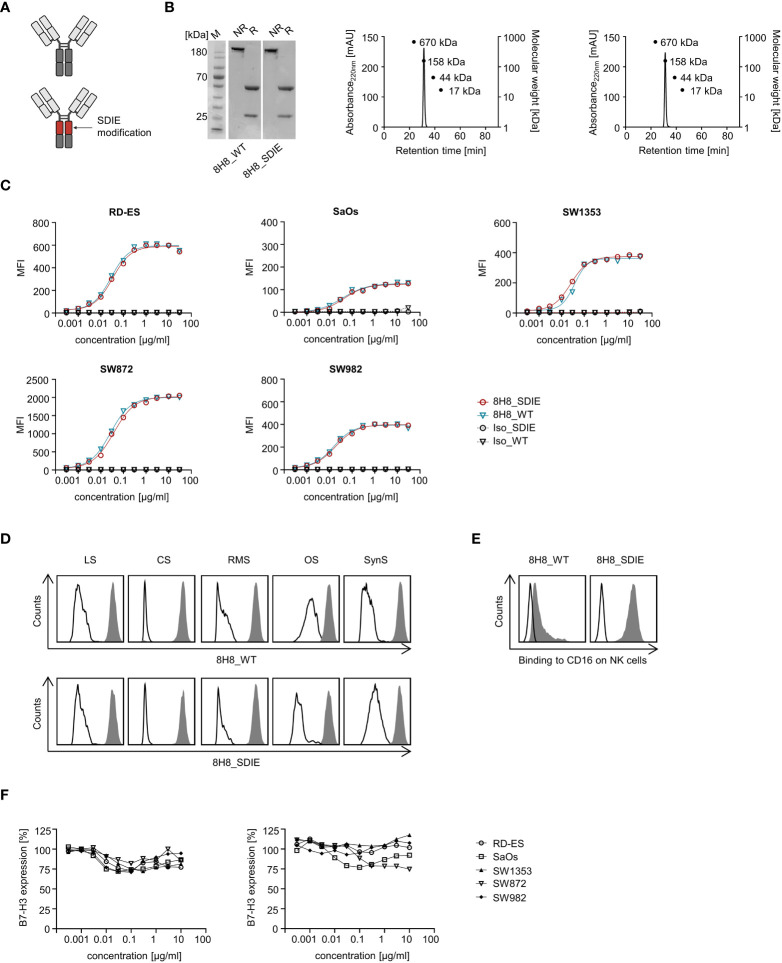
Generation and binding characteristics of B7-H3 specific antibodies. **(A)** Schematic illustration of the generated B7-H3 specific antibodies, either with wildtype Fc part (8H8_WT) (top) or Fc optimized part to enhance affinity to CD16 (8H8_SDIE) (bottom). Created with BioRender.com. **(B)** Exemplary results of an SDS PAGE (left panel) for both B7-H3 antibodies and of size exclusion chromatography for 8H8_WT (middle panel) and 8H8_SDIE (right panel) **(C)** Sarcoma cell lines were incubated with the indicated concentrations of 8H8_WT, 8H8_SDIE or the corresponding isotype controls followed by an anti-human PE conjugate and analyzed by flow cytometry. Exemplary data for mean fluorescence intensity (MFI) levels from one representative experiment of a total of three with similar results are shown. **(D)** Binding of 8H8_WT and 8H8_SDIE or the corresponding controls (1 µg/ml) to the surface of patient-derived sarcoma cells was analyzed by flow cytometry using the 8H8 antibodies (shaded peaks) and the corresponding isotype controls (open peaks), respectively. **(E)** Specific binding of 8H8 mAbs to CD16 on NK cells was analyzed by flow cytometry using NK cells (CD3^-^ CD56^+^) within healthy donor PBMC incubated without (open peaks) or with the 8H8 mAbs (shaded peaks) followed by an anti-human PE conjugate. **(F)** Sarcoma cells RD-ES, SaOs, SW1353, SW872 and SW982 were incubated with indicated concentrations of 8H8_WT, 8H8_SDIE or the corresponding controls for 24 h (left panel) or 72 h (right panel), respectively. Then, cells were washed and reincubated with 1 μg/ml of 8H8_SDIE, followed by an anti-human PE conjugate (1:100) and then analyzed by flow cytometry. Relative surface expression of B7-H3 was calculated by defining the mean fluorescence intensity of cells preincubated without antibody as 100%. Exemplary data from one representative experiment of a total of three are shown.

From a panel of mAbs directed to B7-H3 (described in patent application EP3822288A1), a humanized mAb with suitable binding characteristics (clone 8H8) was selected for generation of our B7-H3-targeting mAbs with either a human IgG1 wildtype Fc part (8H8_WT) or a human IgG1 part, which contains the amino-acid substitutions S239D/I332E (8H8_SDIE) described to increase the affinity to the Fc receptor CD16, which mediates ADCC (**Figure 2A**). As controls served wildtype and Fc optimized mAbs with non-relevant target specificity termed Iso_WT and Iso_SDIE (40). The mAbs were then produced as described in the material and methods section. To biochemically characterize the produced mAbs, SDS-PAGE and gel filtration was conducted and revealed the expected molecular weights for LC, HC, and full mAb, for both 8H8_WT and 8H8_SDIE, and confirmed the lack of aggregates (**Figure 2B**). Next, dose titration experiments (range 3-30,000 ng/ml) with the five different sarcoma cell lines were performed using flow cytometry. We observed saturated binding of both 8H8_WT and 8H8_SDIE at concentrations of about 1000 ng/ml. Specificity and affinity of the B7-H3 mAbs was not affected by the Fc optimization (**Figure 2C**), and the concentration of 1000 ng/ml was used for further analyses. Binding of 8H8_WT and 8H8_SDIE to sarcoma cells was confirmed with samples derived from patients with sarcomas of various subtypes (**Figure 2D**). Next, we analyzed the binding of both mAbs to CD16 on NK cells within resting PBMC. 8H8_WT bound to NK cells, while 8H8_SDIE displayed substantially more pronounced binding (**Figure 2E**; **Supplementary Figures 1, 2**).

Binding of an antibody to its target molecule often results in a dose-dependent modulation of target antigen expression. This in turn impairs therapeutic efficacy (41). Therefore, we analyzed the antigen shift induced by our 8H8_SDIE on sarcoma cells. Upon exposure to different concentrations of 8H8_SDIE (range 3–10 000 ng/ml) for 24 h or 72 h, only marginal reduction of B7-H3 on the cell surface was observed (**Figure 2F**).

### B7-H3-targeting mAbs induce NK cell reactivity against sarcoma cells

**Figure 3:**
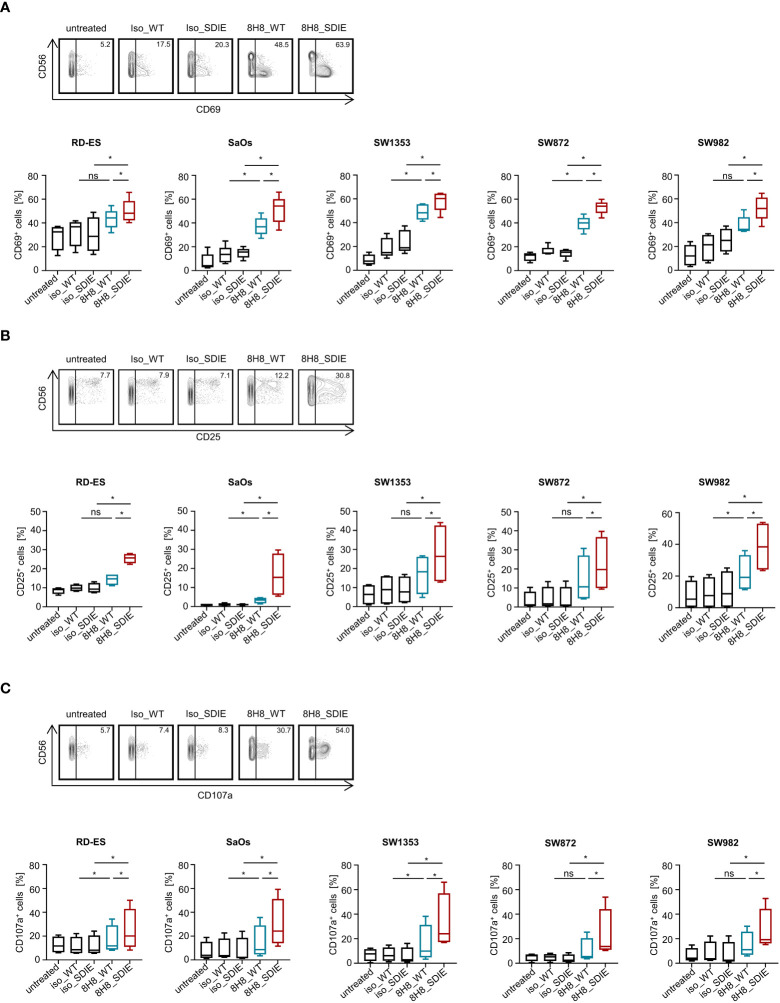
Induction of NK cell reactivity by B7-H3 antibodies against sarcoma cells. PBMC of healthy donors were cultured with or without sarcoma cells at an E:T ratio of 2.5:1 in the presence or absence of B7-H3 antibodies or the corresponding isotype controls (1 μg/mL). **(A)** Activation of NK cells was determined by expression of CD69 after 24h. In the upper panels, exemplary flow cytometry results obtained with SaOs and in the lower panels data for sarcoma cell lines RD-ES, SaOs, SW1353, SW872 and SW982 (n=5) with PBMC of 4 different donors are shown. **(B)** Activation of NK cells was determined by expression of CD25 after 72h. In the upper panels exemplary flow cytometry results obtained with RD-ES and in the lower panels data with sarcoma cell lines RD-ES, SaOs, SW1353, SW872 and SW982 with PBMC of 4 different donors are shown. **(C)** Degranulation of NK cells was determined by expression of CD107a after 4h. In the upper panels exemplary flow cytometry results obtained with SW872 and in the lower panels data with sarcoma cell lines RD-ES, SaOs, SW1353, SW872 and SW982 with PBMC of four independent donors are shown. ns, not significant; *statistically significant differences (p-value < 0.05).

**Figure 4:**
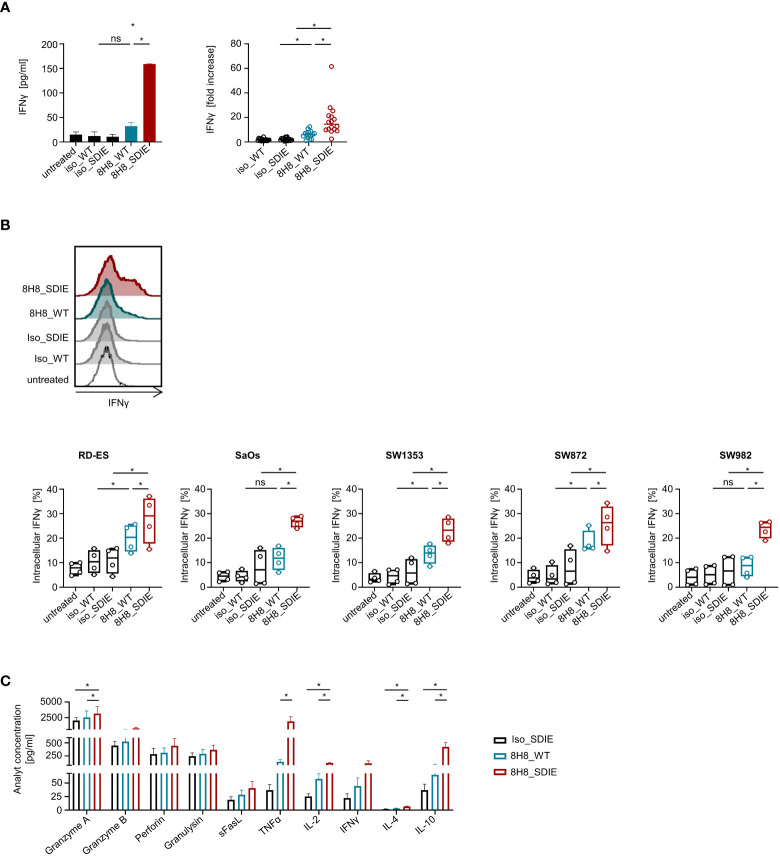
Induction of immunoregulatory molecules by B7-H3 antibodies against sarcoma cells. Healthy donor PBMC were cultured with sarcoma cells at an E:T ratio of 2.5:1 in the presence or absence of 8H8_WT, 8H8_SDIE or the corresponding isotype controls (1 μg/mL) **(A,B)** Supernatants and NK cells within PBMC were analyzed for IFNγ. **(A)** Release of IFNγ after 24 h was analyzed in supernatants of cocultures by Legendplex assays. In the left panel, exemplary results with SW1353 are shown, in the right panel results obtained with sarcoma cell lines RD-ES, SaOs, SW1353 and SW872 and with PBMC of four independent donors are shown. **(B)** Intracellular expression of IFNγ in NK cells within PBMC identified by counterstaining with CD3^-^ CD56^+^ was analyzed after 4 h by flow cytometry. In the upper panel, exemplary results obtained with SaOs and in the lower panel data obtained with sarcoma cells (RD-ES, SaOs, SW1353, SW872 and SW982) and with PBMC of four independent donors are shown. **(C)** Supernatants were analyzed for effector molecules Granzyme A, Granzyme B, Perforin, Granulysin and sFasL and for release of immunoregulatory molecules TNFα, IL-2, IFNγ, IL-4, IL-10 after 4 h by Legendplex assays. Shown are pooled results with sarcoma cell lines SW1353 and SW872 and with PBMC of two independent donors. ns, not significant; *statistically significant differences (p-value < 0.05).

Next, we determined whether and how our B7-H3-targeting mAbs induce NK cell anti-sarcoma reactivity. For these studies, healthy donor PBMC bearing NK cells as immune effector cells were cultivated with sarcoma cell lines with or without 8H8_WT, 8H8_SDIE or the corresponding isotype controls. Analysis of NK cells within PBMC for CD69 expression after 24 h by flow cytometry revealed that 8H8_WT already enhanced NK cell activation, and NK cell activation was further significantly enhanced upon treatment with 8H8_SDIE for all sarcoma cell lines. The control mAbs with nonrelevant target specificity had no significant effect (**Figure 3A**). Since CD25^+^ NK cells exhibit a higher proliferative activity (42), we analyzed induction of the activation marker CD25 on NK cells. CD25 expression was significantly increased upon incubation of PBMC with all sarcoma cell lines for 72 h with 8H8_SDIE, but not with 8H8_WT for cocultures with all cell lines or controls (**Figure 3B**). CD107a serves as surrogate marker for degranulation of NK cells. Flow cytometry analysis of CD107a expression revealed that presence of 8H8_WT in cocultures with sarcoma cells RD-ES, SaOs and SW1353 already significantly enhanced CD107a expression, and a significantly more pronounced effect was observed for all cell lines upon treatment with 8H8_SDIE, whereas control mAbs had no relevant effect (**Figure 3C**). Analysis of IFNγ secretion into culture supernatants of PBMC and sarcoma cell lines by Legendplex assays showed an increase in cytokine release after treatment with 8H8_WT with a significantly higher effect upon incubation with 8H8_SDIE (**Figure 4A**). IFNγ is a cytokine that mediates various immunomodulatory effects including direct anti-tumor effects, but also enables NK cells to shape subsequent adaptive immune responses (43). Analysis by intracellular flow cytometry confirmed that induction of IFNγ was significantly increased in NK cells upon treatment with both 8H8_WT and 8H8_SDIE, with again superior effects of 8H8_SDIE (**Figure 4B**). Finally, we analyzed the release of effector and immunomodulatory molecules mediating NK cell effector functions in supernatants after coculturing PBMC and sarcoma cells using Legendplex assays. The presence of 8H8_WT already enhanced levels of analyzed molecules, whereas a clear tendency for enhanced levels of Granzyme B, Perforin, Granulysin, sFasL and IFNγ was observed for 8H8_SDIE, whereas levels of Granzyme A, TNF, IL-4 and IL-10 were significantly increased (**Figure 4C**).

### Induction of target cell lysis by B7-H3-targeting mAbs 8H8_SDIE and 8H8_WT

**Figure 5:**
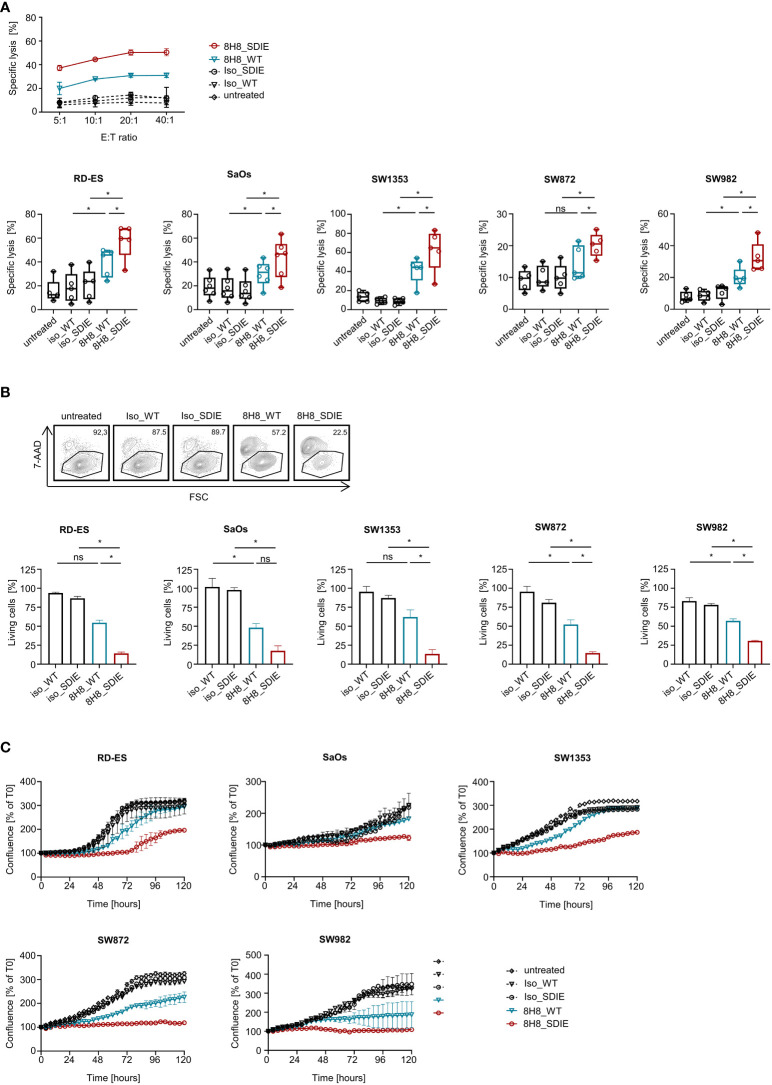
Induction of target cell lysis by Fc optimized B7-H3 antibody. PBMC of healthy donors were incubated with different sarcoma cell lines (n = 5) and treated without or with indicated B7-H3 antibodies or corresponding isotype controls (1 μg/mL). **(A)** Lysis of sarcoma cell lines RD-ES, SaOs, SW1353, SW872 and SW982 (n=5) was analyzed by 2 h Europium cytotoxicity assays. In the top panel, exemplary data obtained with SW982 and one PBMC donor with different E:T ratios and in the bottom panel, pooled data for each cell line obtained with PBMC of healthy donors (n=5) at an E:T ratio of 20:1 are shown. **(B)** Lysis of sarcoma cell lines RD-ES, SaOs, SW1353, SW872 and SW982 (n=5) was determined after 72 h by flow cytometry-based lysis assays at an E:T ratio of 10:1. In the top panel exemplary dot plots with SW872 and one PBMC donor are shown, the bottom panel depicts combined results for each cell line with PBMC (n=5) of healthy donors. **(C)** Cell death of sarcoma cells was determined using a live cell imaging system. Sarcoma cell lines RD-ES, SaOs, SW1353, SW872 and SW982 were incubated with PBMC of two healthy donors at an E:T ratio of 10:1 for 120h. T=0 h corresponds to a confluence of 100%. Results are shown as mean ± SD. ns, not significant; *statistically significant differences (p-value < 0.05).

Next, we investigated whether the enhanced NK cell activity was mirrored in analyses of cytotoxicity. To this end, we determined the capacity of 8H8_SDIE and 8H8_WT to induce target cell lysis by NK cells. Cocultures of bone and soft tissue sarcoma cell lines RD-ES, SaOs, SW1353, SW872 and SW982 with healthy donor PBMC revealed that 8H8_WT enhanced target cell lysis in short-term cytotoxicity assays. Sarcoma cell lysis induced by 8H8_SDIE was clearly superior to 8H8_WT for all different sarcoma cells lines, whereas presence of control mAbs had no effect on target cell lysis (**Figure 5A**). In line, analysis in long-term FACS based lysis assays over 72 h showed pronounced efficacy of 8H8_SDIE against sarcoma cells (**Figure 5B**) compared to 8H8_WT. Despite the heterogenous morphology and growth rates of the different sarcoma cell lines, the superior capacity of 8H8_SDIE to induce target cell lysis compared to 8H8_WT was additionally confirmed in extended analyses of sarcoma cell lysis observed for 120 h by live cell imaging (**Figure 5C**, **Supplementary Figure 3**). Of note, 8H8_WT also showed a clear tendency to induce NK cell reactivity against sarcoma targets, but upon treatment with 8H8_SDIE, profound sarcoma cell lysis was observed with all five cell lines displaying varying levels of B7-H3 on the cell surface.

### B7-H3-targeting mAbs induce NK reactivity against patient-derived sarcoma cells

**Figure 6:**
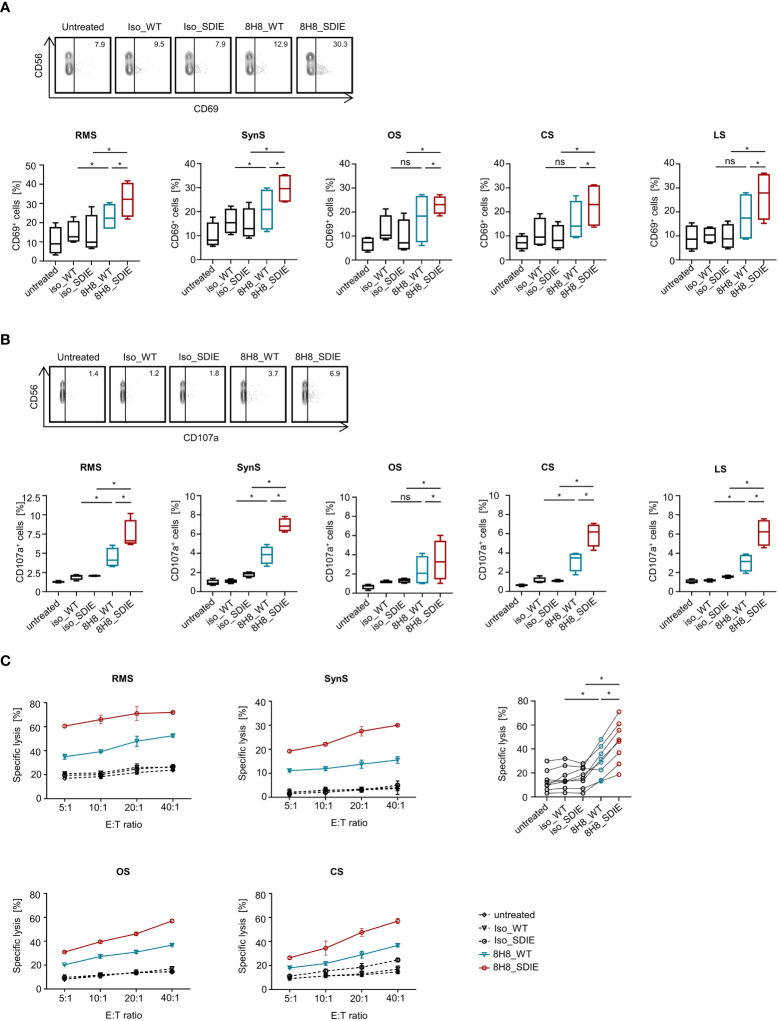
8H8_SDIE induces NK cell reactivity and cytotoxicity against patient-derived sarcoma cells. PBMC of healthy donors were incubated with patient-derived sarcoma cells of different subtypes and treated with the indicated B7-H3 antibodies or the corresponding isotype controls (1 μg/mL). **(A)** Activation of NK cells was determined by expression of CD69 after 24h. In the top panels, exemplary flow cytometry results obtained with patient-derived synovialsarcoma cells and one PBMC donor and in the bottom panel, combined data with patient-derived sarcoma cells (n=5) and with PBMC of four different donors (n=4) are shown. **(B)** Degranulation of NK cells was determined by expression of CD107a after 4h. In the upper panels, exemplary flow cytometry results obtained with patient-derived rhabdomyosarcoma cells and one PBMC donor and in the lower panel, data with patient-derived sarcoma cells (n=5) and with PBMC of four independent donors (n=4) are shown. **(C)** Killing of patient-derived sarcoma cells (n=4) was analyzed by 2 h Europium cytotoxicity assays. In the left panels, exemplary data obtained with PBMC of one healthy donor and patient-derived rhabdomyosarcoma, osteosarcoma, synovialsarcoma and chondrosarcoma cells as indicated with different E:T ratios and on the right, pooled data obtained with PBMC of healthy donors (n=2) and patient-derived sarcoma cells (n=4) at an E:T ratio of 20:1 are shown. ns, not significant; *statistically significant differences (p-value < 0.05).

Finally, we investigated the efficacy of our B7-H3-targeting mAbs in comparative analyses of 8H8_SDIE versus 8H8_WT and respective controls to induce NK reactivity against sarcoma cells derived from patients diagnosed with liposarcoma, chondrosarcoma, rhabdomyosarcoma, osteosarcoma or synovial sarcoma. Cocultures of healthy donor PBMC containing NK cells as effector cells with B7-H3 expressing patient-derived sarcoma cells revealed, alike our flow cytometric analyses with sarcoma cell lines, that treatment with 8H8_WT already enhanced NK cell activation and degranulation (**Figures 6A, B**). Treatment with 8H8_SDIE caused a pronounced and significant increase in activation and degranulation as compared to 8H8_WT, whereas presence of control antibodies Iso_WT and Iso_SDIE had no relevant effect. In line, the findings for 8H8_SDIE on NK cell activation and degranulation resulted in potent induction of ADCC and ultimately tumor cell lysis. Short-term cytotoxicity assays confirmed that treatment with 8H8_WT and 8H8_SDIE induced a clearly target-antigen restricted lysis, whereas 8H8_SDIE induced superior killing as observed with all tested patient-derived sarcoma cells of different origin (**Figure 6C**). Likewise, B7-H3 mAB induced profoundly higher and long-lasting lysis of sarcoma cells. Hence, 8H8_SDIE is able to potently elicit NK cell immunity against primary sarcoma cells regardless of their subtype.

## Discussion

Therapeutic modalities for sarcoma patients have improved in recent years, yet treatment and especially cure of sarcomas remains a challenge. So far, FDA-approved antibody-based approaches in sarcoma like PD-1/PDL-1 checkpoint inhibition (Dostarlimab and Pembrolizumab) or Denosumab for treatment of a subset of bone sarcomas are restricted to a minority of patients (8). Accordingly, new therapeutic concepts are urgently needed. Interestingly, sarcomas were already treated with Coley’s toxins about 100 years ago, the latter in retrospective being a precursor of modern immunotherapy (44).

In the presented study, we report the preclinical characterization of an Fc-optimized B7-H3-targeting mAb termed 8H8_SDIE for treatment of sarcoma. Sarcoma cell lines of different origin as well as primary sarcoma cells from patients diagnosed with soft-tissue and bone sarcomas were identified to express substantial quantities of B7-H3. Our optimized mAb 8H8_SDIE showed optimal binding characteristics with all sarcoma cells and NK effector cells. No relevant downregulation of B7-H3 expression (antigen shift) that can impair efficacy of mAb treatment (41) was observed upon incubation with 8H8 mAbs. The anti-tumor activity induced by 8H8_SDIE against sarcomas was superior to its counterpart with a wildtype Fc part, and this was confirmed in multiple experimental settings using sarcoma cell lines as well as patient-derived sarcoma cells.

An important subset of cytotoxic lymphocytes are NK cells, which largely contribute to cancer immune surveillance. Their efficacy is based on their ability not only to mediate direct cytotoxicity, but also to influence subsequent immune responses of the adaptive immune system. Accordingly, numerous attempts are currently aiming at using NK cells for treatment of cancer (45, 46). Application of antitumor antibodies which induce ADCC represents a promising therapeutic approach for many cancers, as demonstrated for example by the clinical success of rituximab. The effect of the latter mAb is mainly based on induction of ADCC. Meanwhile, rituximab is well established for treatment of various B cell malignancies (47). To further enhance ADCC induced by therapeutically utilized antibodies and thus to increase efficacy, several strategies currently aim at generation of improved antitumor mAbs using the approach of Fc-optimization to increase affinity for CD16. Besides modifying glycosylation motifs (15), increased affinity to CD16 can also be attained by changing the amino acid sequence in the CH2 domain of the Fc part for example by the S239D/I332E substitutions (SDIE modification) (14) that is also contained in our B7-H3-targeting mAb 8H8_SDIE. The Fc optimization resulted in a significant increase in NK-mediated ADCC against sarcoma cells as compared to 8H8_WT that contains a wildtype Fc part. At present, many Fc-optimized mAbs that comprise the SDIE modification successfully undergo clinical evaluation, for example FLYSYN (anti-FLT3; NCT02789254), margetuximab (anti-HER2; NCT01828021), BI 836858 (anti-CD33; NCT02240706, NCT03013998), and MEN1112 (anti-CD157; NCT02353143) or FDA approved tafasitamab (anti-CD19).

In previous studies, we evaluated various mAbs and fusion proteins containing the SDIE modification for improved induction of ADCC, e.g. in leukemia, colorectal cancer, breast cancer as well as sarcoma, some of them until the stage of clinical application (34, 35, 38, 40, 48–51). Here we set out to develop an B7-H3 directed Fc-optimized mAb for treatment of sarcomas based on the reasoning that B7-H3 is reportedly overexpressed in many solid tumors (23, 25), whereas expression in healthy tissues is limited (39, 52), and that sarcomas are NK cell-sensitive cancer types (45). Our efforts were further prompted by our observation that B7-H3 is overexpressed in bone and soft tissue sarcomas independently of subtype, as tumor associated antigens with homogenous overexpression are a prerequisite for success of immunotherapeutic treatment. Of note, in some recent reports the B7-H3 protein has been characterized as a checkpoint molecule that exerts immunosuppressive and tumor promoting activity (53, 54). There is also first evidence that the B7-H3 positive cell fraction within cancer cells potentially represents cancer stem cells (55–57). Based on these findings, it is not surprising that multiple immunotherapeutic approaches directed against B7-H3 are currently under clinical investigation. This includes, but is not limited to strategies like antibody-drug conjugates (MGC018: NCT03729596; DS7300a: NCT04145622), Fc-optimized mAbs (MGA271, enoblituzumab: NCT02923180, NCT02475213, NCT04634825; DS-5573a: NCT02192567, clinical trial terminated), radiolabeled mAbs (^131^I-8H9: NCT03275402, NCT04022213; ^177^Lu-DPTA omburtamab: NCT04315246, NCT04167618), and bispecific antibodies (MGD009: NCT026285351, clinical trial terminated) (58). Our work expands this armamentarium to Fc-optimized mAbs for potent induction of NK cell ADCC.

Regarding toxicity/side effects expected by targeting B7-H3, it must be considered that B7-H3 is not only (inducible) expressed on antigen-presenting cells (16), but basal expression is also reported for endothelial cells, resting fibroblasts, amniotic fluid stem cells and osteoblasts (52, 59). However, in preclinical studies which used B7-H3 as therapeutic target including B7-H3-targeting chimeric antigen receptor (CAR) T cells, significant anti-tumor effects in preclinical models (23, 26, 27, 60, 61), but no toxicity was observed, likely due to profoundly lower B7-H3 antigen levels in healthy tissue (27). In line, the first evaluations of B7-H3-targeting immunotherapeutics in clinical studies like anti-B7-H3 antibodies and B7-H3 CAR-T cells did not reveal any unbearable toxicity and off-tumor effects against healthy B7-H3 expressing cells (58, 62). Nevertheless, this issue requires further elucidation.

In conclusion, 8H8_SDIE showed powerful anti-sarcoma effects in a preclinical setting. Of note, our treatment approach was not restricted to a distinct sarcoma entity, as different sarcoma cells lines as well as patient-derived sarcoma cells including osteosarcoma, rhabdomyosarcoma, synovial sarcoma liposarcoma and chondrosarcoma were sensitive to treatment with our B7-H3-targeting mAbs. 8H8_SDIE could thus constitute an immunotherapeutic option for sarcoma patients. Although future studies including *in vivo* experiments are certainly warranted to fully characterize 8H8_SDIE, the data presented in this study underscore the potential of our B7-H3-targeting Fc-optimized mAb for sarcoma treatment.

## Data availability statement

The original contributions presented in the study are included in the article/**Supplementary Material**. Further inquiries can be directed to the corresponding author.

## Ethics statement

The studies involving human participants were reviewed and approved by IRB (ethics committee of the Faculty of Medicine of the Eberhard Karls Universitaet Tuebingen and of the University Hospital Tuebingen) and was conducted in accordance with the Declaration of Helsinki; reference number 13/2007V and 612/2010BO2. The patients/participants provided their written informed consent to participate in this study.

## Author contributions

IH designed and performed the experiments, analyzed and interpreted data, and wrote the manuscript. ME designed and performed experiments. TM provided the B7-H3 mAb. MM contributed to the study design and contributed to writing of the manuscript. CH provided patient samples and contributed to writing of the manuscript, GJ contributed to study design and writing of the manuscript. HS contributed to the study design, critically revised the manuscript, and co-supervised the study. LZ designed and supervised the study and contributed to writing of the manuscript. All authors approved the submitted version of the manuscript.

## Funding

This project was supported by the Deutsche Krebshilfe (70113999, 70114180), Wilhelm Sander-Stiftung (2017.100.2), and Deutsche Forschungsgemeinschaft (DFG, German Research Foundation) under Germany’s Excellence Strategy - EXC 2180 - 39090067 and DFG, project number SA 1360/9-3).

## Acknowledgments

The authors thank Andrea Dobler und Carolin Walker for excellent technical assistance. Flow cytometry sample acquisition was performed on shared instruments of the Flow Cytometry Core Facility Tuebingen.

## Conflict of interest

GJ, HS, LZ, and TM are listed as inventors on the patent application “Antibodies targeting, and other modulators of, the CD276 antigen, and uses thereof,” EP3822288A1, applicant German Cancer Research Center, Heidelberg, Germany.

The remaining authors declare that the research was conducted in the absence of any commercial or financial relationships that could be construed as a potential conflict of interest.

## Publisher’s note

All claims expressed in this article are solely those of the authors and do not necessarily represent those of their affiliated organizations, or those of the publisher, the editors and the reviewers. Any product that may be evaluated in this article, or claim that may be made by its manufacturer, is not guaranteed or endorsed by the publisher.
