# Supplementary material for: B7-H3-targeting Fc-optimized antibody for induction of NK cell reactivity against sarcoma
Source: Front Immunol. 2022 Oct 7;13:1002898. doi: 10.3389/fimmu.2022.1002898 (PMC9585277; doi:10.3389/fimmu.2022.1002898)
Supplement: Supplementary file 1 [file DataSheet_1.docx]

Supplementary Material

# Supplementary Figures

#
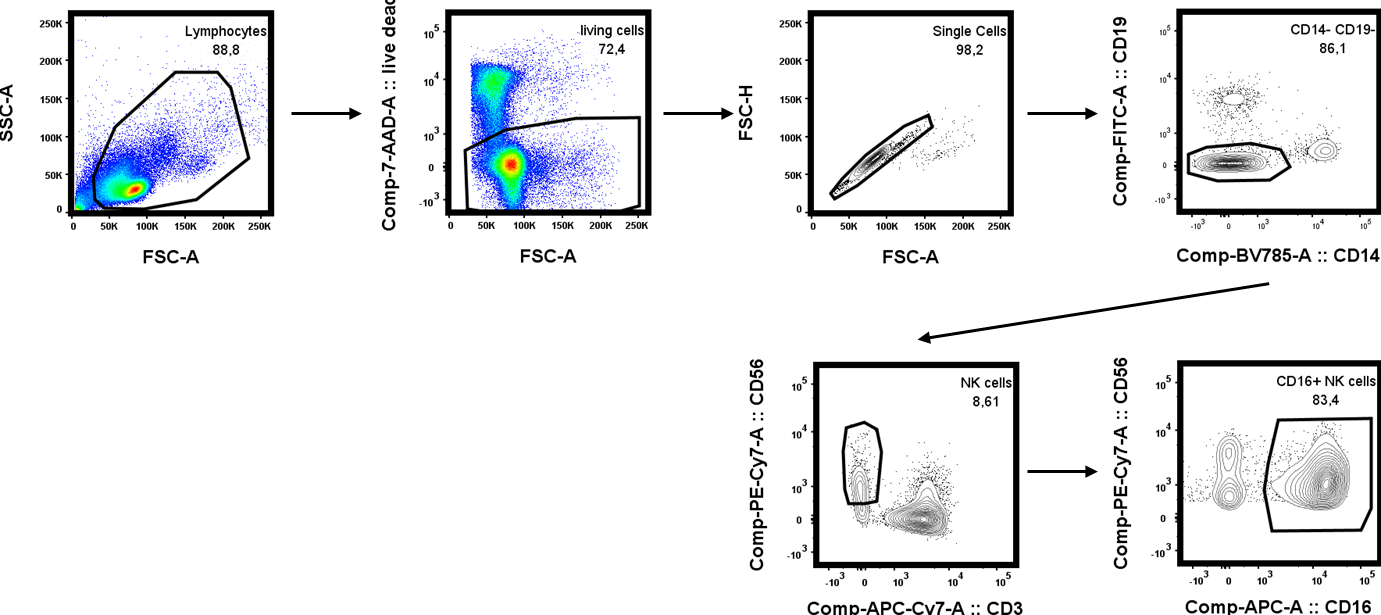


# Supplementary Figure 1. Representative gating strategy (Lymphocytes -> 7-AAD^-^ living cells -> single cells -> CD14^-^ CD19^+^ cells -> CD3^-^CD56^+^ NK cells -> CD16) for NK cells within healthy donor PBMC.

**
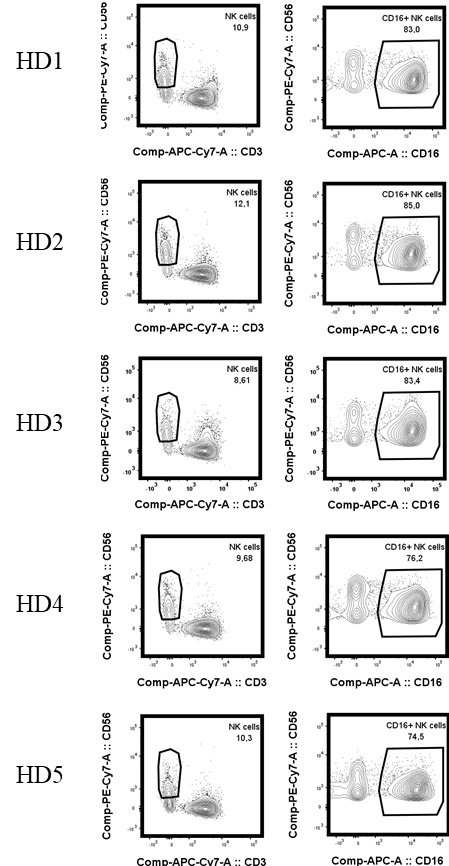
**

**Supplementary Figure 2.** Exemplary results for NK cells within healthy donor (HD) PBMC and CD16 expression on NK cells for n=5 HD PBMC.


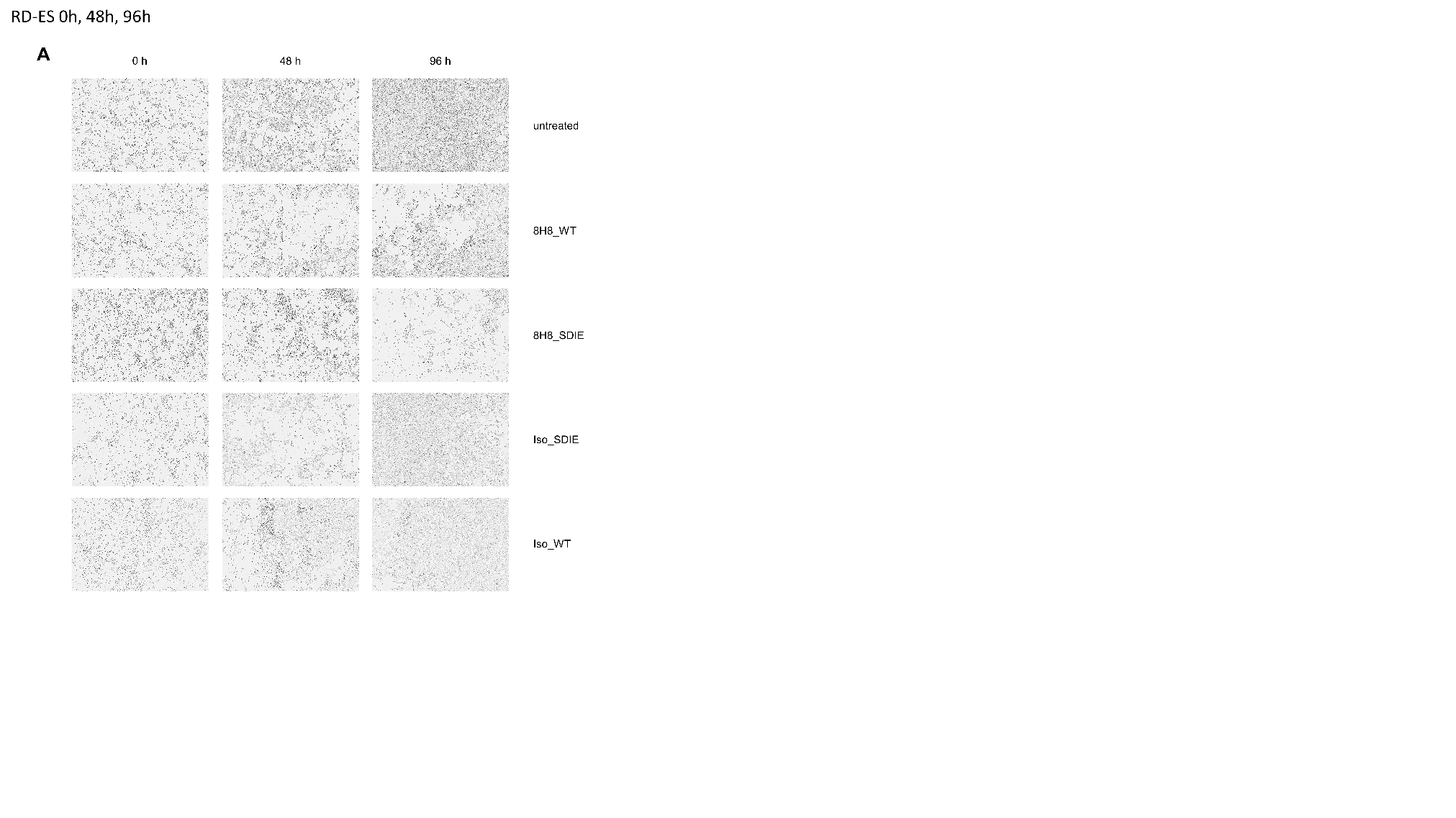


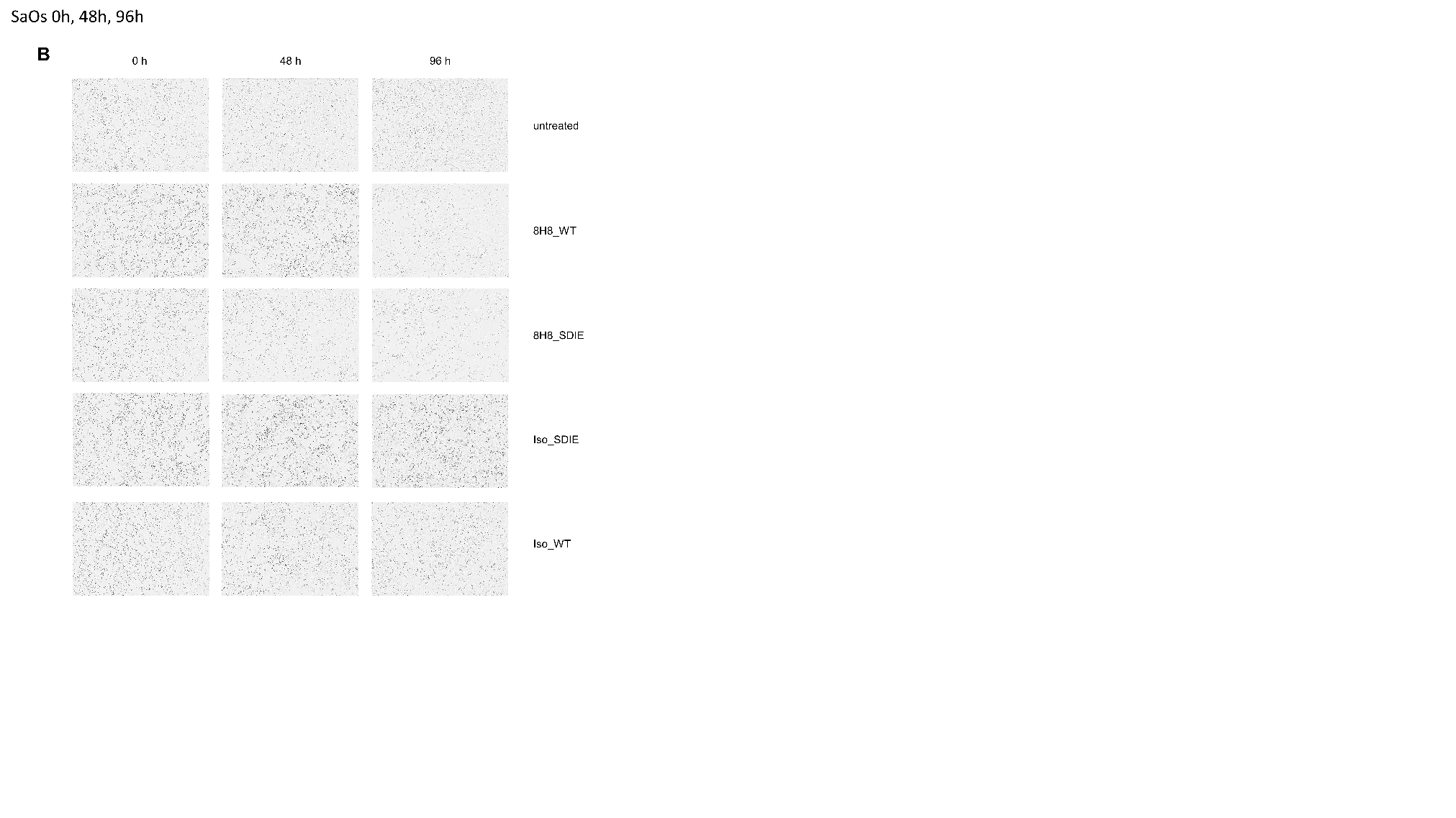


**Supplementary Figure 3.** Cell death of sarcoma cells was determined using a live cell imaging system. Cells were incubated with PBMC of healthy donors at an E:T ratio of 10:1 for 120 h. Representative images at 0, 24, and 96 h for cell lines RD-ES (**A**) and SaOs (**B**) with one PBMC donor are displayed. 10x magnification.
